# Evaluation of an In-House Developed Foot-and-Mouth Disease Virus SAT 3 Vaccine Strain Based on Antigen Productivity and Inactivation Kinetics for Commercial Feasibility

**DOI:** 10.3390/vaccines14050381

**Published:** 2026-04-24

**Authors:** Jae Young Kim, Sun Young Park, Gyeongmin Lee, Giyoun Cho, Seung-A Hwangbo, Jong-Hyeon Park, Young-Joon Ko

**Affiliations:** Center for Foot-and-Mouth Disease Vaccine Research, Animal and Plant Quarantine Agency, 177 Hyeoksin 8-ro, Gimcheon-si 39660, Republic of Korea; ivorikim@korea.kr (J.Y.K.); sun3730@korea.kr (S.Y.P.); lgm6004@korea.kr (G.L.); libretto@korea.kr (G.C.); hbsa230@korea.kr (S.-A.H.); parkjhvet@korea.kr (J.-H.P.)

**Keywords:** foot-and-mouth disease, vaccine, SAT 3 ZIM-R, antigen productivity, inactivation kinetics

## Abstract

Background: In the Republic of Korea, a bivalent foot-and-mouth disease (FMD) vaccine covering serotypes O and A is administered to livestock, while antigens for the other serotypes are stockpiled in overseas antigen banks. To achieve self-reliance in FMD vaccine production, various vaccine strains have been developed using in-house technology. Although SAT 3 has historically been confined largely to Africa, preparedness against this serotype remains necessary, as the possibility of its introduction into Korea cannot be completely excluded. Methods: In this regard, we evaluated the commercial potential of the SAT 3 ZIM-R vaccine strain by assessing antigen productivity, scalability, inactivation kinetics, and immunogenicity. Results: Supplementation with 3 mM Ca^2+^ markedly increased antigen yield compared with that obtained in the absence of calcium. Further optimization showed that antigen yield was highest at pH 8.0–8.5. During scale-up, antigen yield was maintained at 9.2–9.8 μg/mL in flask cultures and remained high at approximately 7.8 μg/mL in a bioreactor, demonstrating robust scalability. Treatment with 2 mM binary ethylenimine at 26 °C achieved complete inactivation within 24 h. Vaccines formulated with the SAT 3 ZIM-R antigen produced either in flasks or in a bioreactor induced comparable neutralizing antibody responses in pigs following both the primary and booster immunizations. Conclusions: Collectively, these findings indicate that SAT 3 ZIM-R is a promising vaccine candidate for large-scale vaccine antigen production and the future establishment of a domestic FMD antigen bank in Korea.

## 1. Introduction

Foot-and-mouth disease (FMD) is a highly contagious transboundary disease of cloven-hoofed animals that causes substantial economic losses through reduced productivity and restrictions on livestock trade [1,2]. The causative agent, FMD virus (FMDV), is a positive-sense, single-stranded RNA virus classified within the genus *Aphthovirus* of the family *Picornaviridae* and comprises seven immunologically distinct serotypes, namely O, A, C, Asia1, Southern African Territories (SAT) 1, SAT 2, and SAT 3 [2].

Its capsid is composed of four structural proteins, VP1, VP2, VP3, and VP4, which assemble into protomers, pentamers, and finally intact virions. The intact 146S particle is regarded as the most relevant antigenic component for inactivated vaccine production because it is much more immunogenic than dissociated capsid forms such as 12S particles [3,4,5]. For this reason, the amount of 146S antigen is a key indicator of vaccine antigen quality and manufacturing efficiency.

Since the nationwide large-scale FMD outbreaks in 2010–2011, Korea has implemented routine vaccination of cattle, pigs, and goats with a bivalent vaccine containing serotypes O and A. In contrast, vaccine antigens against serotypes not included in the domestic vaccination program have been stored in overseas FMD vaccine manufacturing facilities as an antigen bank [6,7]. As a domestic FMD vaccine production facility is being prepared, there is an increasing need to identify suitable vaccine strains that can support the future establishment of a national antigen bank. Because cross-protection among serotypes is limited, vaccine preparedness depends on securing antigenically appropriate strains and maintaining vaccine antigens that can be rapidly deployed in the event of an outbreak [1,2].

SAT 3 has historically been confined largely to Africa [8], but its epidemiological significance cannot be overlooked in a global livestock trading environment. Although SAT 3 has not been reported in Korea, preparedness against possible future incursion is warranted as animal movements and trade can facilitate long-distance spread.

A recombinant SAT 3 vaccine strain, SAT 3 ZIM-R, has previously been generated and shown to confer protective immunity in pigs [9], indicating that it has potential as a vaccine seed strain. Nevertheless, protective efficacy alone is not sufficient for practical use in an antigen bank. For deployment in vaccine manufacturing, the strain must also exhibit high antigen productivity under culture conditions, maintain acceptable performance during scale-up, and tolerate chemical inactivation with minimal loss of structurally intact antigen.

In this study, we aimed to determine whether SAT3 ZIM-R possesses the antigen productivity, inactivation kinetics, and immunological properties required of a practical FMD antigen-bank candidate suitable for commercial vaccine manufacturing.

## 2. Materials and Methods

### 2.1. Cells and Viruses

Suspension-adapted baby hamster kidney cells (BHK-21), which were developed by the Animal and Plant Quarantine Agency (APQA) and the Korea Research Institute of Bioscience and Biotechnology, were maintained in CD BHK-21 Production medium (Gibco, Waltham, MA, USA) at 110 rpm in a 37 °C, 5% CO_2_ shaking incubator. Cell density and viability were analyzed by trypan blue exclusion assay using an automated cell counter (Vi-Cell XR; Beckman Coulter, Brea, CA, USA). Adherent BHK-21 cells (C-13, ATCC CCL-10, Manassas, VA, USA) and Porcine kidney (LFBK) cells (Plum Island Animal Disease Center, Orient, NY, USA) were maintained in DMEM (Corning, NY, USA) supplemented with 10% fetal bovine serum (FBS; Gibco, Paisley, UK) and 1% antibiotic-antimycotic solution (Gibco, Waltham, MA, USA).

The SAT 3 ZIM-R virus used in this study was a recombinant strain generated by reverse genetics through replacement of the structural protein-coding region of an FMDV O1 Manisa full-length infectious clone with the corresponding region of SAT 3 ZIM 4/81 (KX375417) as previously described [9]. Given the intrinsic characteristics of FMDV, all experimental procedures involving its handling are strictly conducted within a biosafety level 3 containment facility.

### 2.2. Establishment of Optimal Infection Conditions for Antigen Generation

Antigen production conditions were systematically evaluated using suspension BHK-21 cultures. Cells were first expanded to high density (~3 × 10^6^ cells/mL) following an initial seeding at 3 × 10^5^ cells/mL and a cultivation period of approximately 3–4 days. Prior to infection, the culture medium was completely refreshed to ensure consistent nutrient availability. Virus inoculation was performed across a range of multiplicities of infection (MOIs), while calcium chloride (Sigma-Aldrich, St. Louis, MO, USA) was introduced at a final concentration of 3 mM concurrently with infection. Cultures were maintained under controlled shaking conditions at 37 °C in a 5% CO_2_ environment. Samples were collected at 12, 16, 20, and 24 h post-infection (hpi) and clarified to remove cellular residues. To further assess physicochemical influences on antigen production, independent cultures were adjusted to predefined pH levels (6.0–9.0) immediately before viral exposure. Following infection at an MOI of 0.01, cultures were incubated for 24 h and processed for virus titration and 146S antigen quantification.

### 2.3. Virus Titration

The infectious capacity of viral samples was determined using a cell-based endpoint dilution assay in BHK-21 monolayers. Serial dilutions were prepared and evaluated in triplicate, and infectious titers were calculated using the Spearman–Kärber statistical approach, with results expressed as TCID_50_ per milliliter [10].

### 2.4. Quantification of FMDV Particles

To minimize analytical interference from host cell-derived components, clarified culture fluids were subjected to chloroform (Merck KGaA, Darmstadt, Germany) extraction prior to analysis. Following phase separation by centrifugation, the aqueous fraction was recovered and treated with Benzonase (Sigma-Aldrich) to degrade residual nucleic acids. Quantification of intact virions was subsequently performed using size-exclusion chromatography (Agilent Technologies, Santa Clara, CA, USA). Separation was achieved on a gel-filtration column (TSKgel G4000PWXL, TOSOH Bioscience, Tokyo, Japan) under defined buffer conditions (Tris-HCl/NaCl, pH 8.0), and elution profiles were monitored to identify peaks corresponding to 146S particles. Antigen concentrations were calculated based on integrated peak areas using the OpenLAB CDS ChemStation software (Version 3.2.0.620) according to a previous study [11].

### 2.5. Production of Vaccine Antigen at Different Culture Scales

To assess scalability, antigen production was examined in both shake-flask and bioreactor systems. In flask-based experiments, suspension BHK-21 cells were seeded at 3 × 10^5^ cells/mL in working volumes of 40, 200, and 1000 mL using Erlenmeyer cell culture flasks (Corning) and grown for 3.5 days to reach about 3 × 10^6^ cells/mL. The cells were then infected with SAT 3 ZIM-R under the optimized infection conditions identified in the preliminary experiments (0.01 MOI, pH 8.0). Supernatants were collected after 24 h and clarified prior to analysis.

For bioreactor production (1000 mL), suspension BHK-21 cells were grown in a 2 L bioreactor (Sartorius, Göttingen, Germany) equipped with automatic control systems for temperature, dissolved oxygen, and pH. The culture was maintained at 37 °C with dissolved oxygen controlled at 45% air saturation, and agitation was set at 150 rpm. Culture pH was regulated using CO_2_ and sodium hydroxide. Once the target cell density was achieved, the culture temperature was lowered to 4 °C, and agitation, pH control, and air supply were discontinued. The cells were then allowed to settle by gravity for at least 12 h. Subsequently, 90% of the total culture volume was replaced with fresh medium, and the pH was adjusted to pH 8.0 before infection with SAT 3 ZIM-R at 0.01 MOI. Supernatants were harvested at 24 hpi and processed identically to flask-derived samples.

### 2.6. FMDV Inactivation Kinetics

Binary ethylenimine (BEI) was freshly synthesized prior to its use through alkaline conversion of bromoethylamine hydrobromide (Sigma-Aldrich). Virus-containing supernatants were exposed to varying BEI concentrations (0.5–3 mM) and incubated at controlled temperatures (26 °C or 37 °C) with continuous mixing. Aliquots were collected every hour during the first 6 h and additionally at 24 h to assess residual infectivity in triplicate during the inactivation process. Chemical inactivation was immediately terminated in each sample through the addition of sodium thiosulfate (Daejung Chemicals & Metals, Siheung, Republic of Korea).

### 2.7. Animal Experiment

Purified SAT 3 ZIM-R antigens obtained from either flask-based or bioreactor-based production systems were formulated as monovalent vaccine candidates, each containing 15 µg of inactivated virus per dose. The vaccine formulation included ISA 206 VG adjuvant (Seppic, Paris, France) at a 1:1 (*v*/*v*) ratio, supplemented with 1% saponin (Sigma-Aldrich) and 1% aluminum hydroxide gel (General Chemical, Mount Laurel, NJ, USA), and adjusted to a final volume of 2 mL per dose. Prepared formulations were incubated in a water bath at 20 °C for 1 h under light-protected conditions and subsequently stored at 4 °C until use. Five seronegative pigs (two months old) were assigned to groups receiving either flask-derived or bioreactor-derived antigens and were immunized intramuscularly twice at a 4-week interval. An additional three pigs were maintained as unvaccinated controls. Blood samples were collected weekly following vaccination and continued until one month after booster immunization. All animal procedures complied with institutional guidelines and were approved by the Animal and Plant Quarantine Agency (APQA; approval no. 2025-1801).

### 2.8. Virus Neutralization Test

Virus neutralization (VN) assays were conducted in duplicate following the guidelines of the WOAH Terrestrial Manual. Serum samples were heat-treated to inactivate complement activity and serially diluted prior to incubation with a fixed dose of virus. Each well received 100 TCID_50_ of SAT 3 ZIM-R virus and was incubated at 37 °C for 1 h. Subsequently, 50 µL of LFBK cell suspension (0.5 × 10^6^ cells/mL) was added. Plates were incubated at 37 °C in a 5% CO_2_ atmosphere for 2–3 days. Neutralizing titers were defined based on the highest serum dilution capable of completely inhibiting infection by 100 TCID_50_ of virus and were expressed in log_10_ values.

### 2.9. Statistical Analysis

Experimental data were generated from independent replicates and summarized as mean values with associated variability. Statistical comparisons between experimental groups were performed using two-way ANOVA implemented in GraphPad Prism software (version 10, La Jolla, CA, USA). A significance threshold of *p* < 0.05 was applied throughout the analysis.

## 3. Results

### 3.1. Optimization of Conditions for SAT 3 ZIM-R Antigen Production

**Figure 1:**
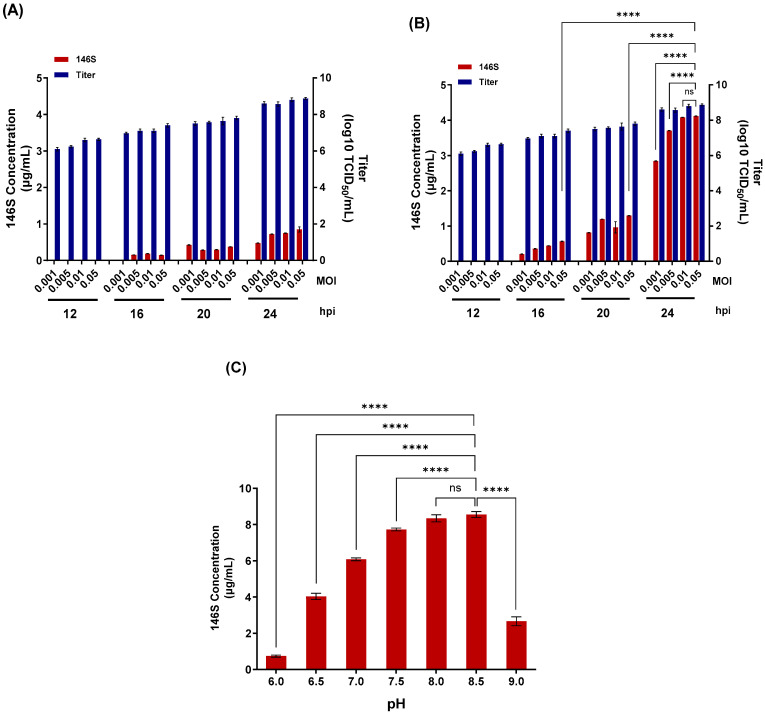
Optimization of SAT3 ZIM-R antigen production under varying virus inoculation conditions. (**A**,**B**) Virus titers and 146S antigen levels were quantified following infection with SAT3 ZIM-R at the indicated multiplicities of infection (MOI) and harvest times (hpi), either in the absence of CaCl_2_ (**A**) or in the presence of 3 mM CaCl_2_ (**B**). (**C**) Antigen yield was further evaluated following adjustment of the culture medium to the indicated pH values at the time of virus inoculation. Data are presented as mean ± standard deviation. Statistical significance is indicated as **** *p* < 0.0001; ns, not significant.

In the absence of calcium, virus titers at 24 hpi ranged from 7.2 to 7.6 log10 TCID_50_/mL depending on the inoculation dose, whereas antigen yields were only 0.47–0.85 μg/mL (Figure 1A). In contrast, when 3 mM CaCl_2_ was added during the virus inoculation step, virus titers increased to 8.6–8.9 log10 TCID_50_/mL at 24 hpi, and antigen yields increased markedly to 2.84–4.12 μg/mL (Figure 1B). Under these conditions, optimal production was achieved at 0.01–0.05 MOI at 24 hpi, yielding up to 4.1 μg/mL of antigen. To further optimize antigen production, the pH of the culture medium was adjusted from 6.0 to 9.0 at the time of virus inoculation in a 40 mL volume. Antigen yields were 0.7 μg/mL at pH 6.0, 4.0 μg/mL at pH 6.5, 6.1 μg/mL at pH 7.0, 7.7 μg/mL at pH 7.5, 8.3 μg/mL at pH 8.0, and 8.5 μg/mL at pH 8.5, followed by a marked decrease at pH 9.0 (Figure 1C). Because there was no significant difference between pH 8.0 and pH 8.5, the optimal pH range for SAT 3 ZIM-R antigen production was considered to be pH 8.0–8.5.

### 3.2. Antigen Yield During Scale-Up of SAT 3 ZIM-R

**Figure 2:**
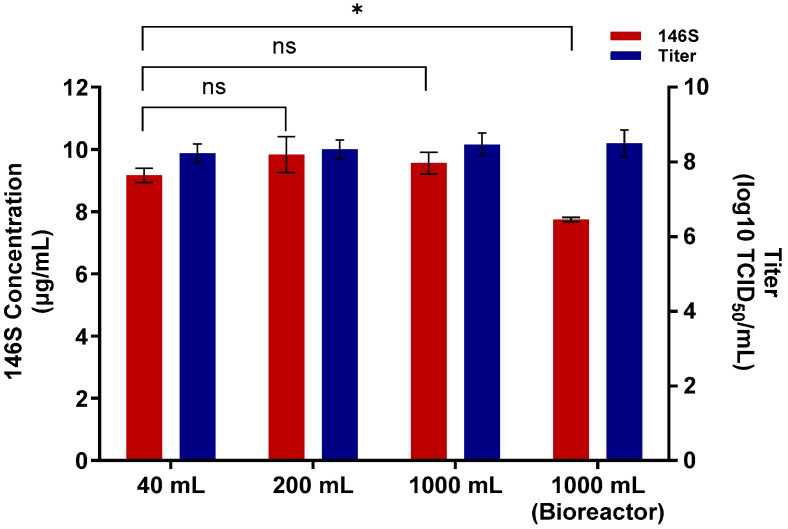
Antigen production efficiency of SAT3 ZIM-R during process scale-up. Virus titers and 146S antigen yields were evaluated across flask cultures with working volumes of 40, 200, and 1000 mL, and compared with those obtained from a bioreactor operated under optimized production conditions. Data are presented as mean ± standard deviation. Statistical significance is indicated as * *p* < 0.05; ns, not significant.

Antigen yield was evaluated at the flask scale using working volumes of 40, 200, and 1000 mL. Virus titers at 24 hpi ranged from 8.2 to 8.5 log10 TCID_50_/mL. Across the different flask volumes, antigen yields were 9.2, 9.8, and 9.6 μg/mL, respectively (Figure 2), indicating stable antigen yield during scale-up at the flask level. For bioreactor culture, SAT 3 ZIM-R was inoculated after the culture medium pH was adjusted to 8.0. The antigen yield was 7.8 μg/mL (Figure 2). These results indicate that high antigen productivity was maintained after transfer to the bioreactor system.

### 3.3. Inactivation Kinetics of the SAT 3 ZIM-R

**Figure 3:**
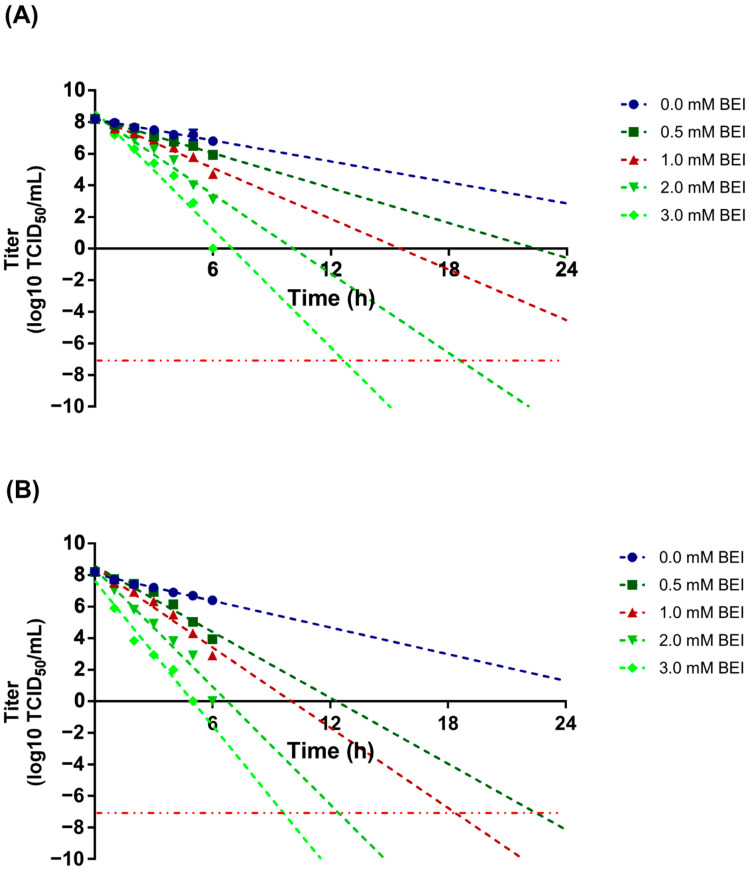
Inactivation kinetics of SAT 3 ZIM-R under different BEI treatment conditions. Virus-containing supernatants were incubated with the indicated concentrations of binary ethylenimine (BEI), and residual infectivity was monitored over time at 26 °C (**A**) or 37 °C (**B**). Residual virus infectivity was measured at designated time points, and decay trends were modeled to estimate the time required to reach the target inactivation threshold (−7 log10 TCID_50_/mL), indicated by the dashed horizontal reference line.

The inactivation conditions for SAT 3 ZIM-R were optimized using virus-containing supernatants obtained from a 2 L bioreactor culture. At 26 °C, complete inactivation below the detection limit was achieved within 24 h at 2 mM BEI. At 37 °C, complete inactivation within 24 h was achieved at 0.5 mM BEI and above (Figure 3). To evaluate antigen loss during inactivation, antigen content was compared between BEI-treated and untreated samples. Under the 26 °C and 2 mM BEI condition, antigen content decreased by 15.7%; however, the untreated control also showed an 11.3% decrease, indicating that the net antigen loss attributable to BEI treatment was only 4.4%. Under the 37 °C and 0.5 mM BEI condition, antigen content decreased by 31.3%, but a 29.6% reduction was observed in the untreated control, suggesting that the loss was caused mainly by heat exposure rather than by the inactivating agent itself. Taken together, these results indicate that the optimal inactivation condition for SAT 3 ZIM-R was treatment with 2 mM BEI at 26 °C (Table 1).

### 3.4. Immunogenicity of the SAT 3 ZIM-R Antigens in Pigs

**Figure 4:**
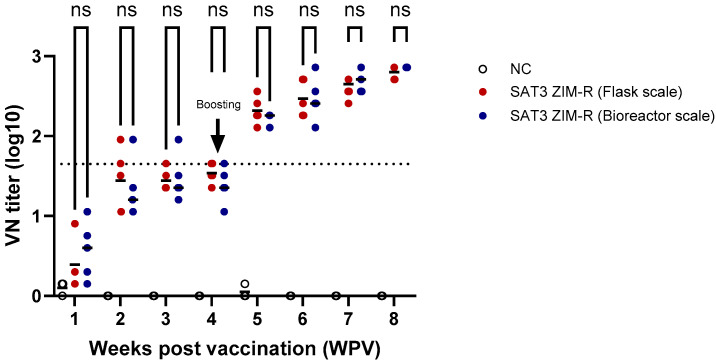
Comparison of antibody responses induced by SAT 3 ZIM-R antigens produced in flasks and in a bioreactor. Pigs were immunized with experimental vaccines prepared from SAT 3 ZIM-R antigens generated in the two production systems, and serum samples were analyzed over time. A virus-neutralizing (VN) titer of >1.65 log was defined as positive, as indicated by the dotted line. The short horizontal line indicates the mean value. Red and blue circles represent antibody titers induced by antigens produced in flasks and in a bioreactor, respectively. ns, not significant.

To compare the immunogenicity of antigens produced by different culture systems, experimental vaccines were prepared using the SAT 3 ZIM-R antigen produced either by flask-scale culture or by bioreactor culture. Pigs seronegative for FMDV were selected, and blood samples were collected once weekly for a total of eight weeks. A booster immunization was administered at 28 days post-vaccination (dpv). At 28 days after the first vaccination, the mean virus-neutralizing antibody titer was 1.54 log10 in the flask-derived antigen group and 1.38 log10 in the bioreactor-derived antigen group. At 28 days after the second vaccination, the mean neutralizing antibody titers increased to 2.80 log10 and 2.95 log10, respectively. No statistically significant difference was observed between the two groups, regardless of the number of immunizations (Figure 4). These findings indicate that the antigen produced in the bioreactor retained immunogenicity comparable to that of antigen produced at the flask scale.

## 4. Discussion

In Korea, all susceptible cloven-hoofed livestock are routinely vaccinated with a bivalent FMD vaccine containing serotypes O and A. By contrast, serotypes not included in the domestic vaccination program, such as Asia1, SAT 1–3, and C, are stored in overseas antigen banks for emergency preparedness. Because a domestic FMD vaccine manufacturing facility is planned, foreign antigen banks are expected to be replaced by a domestic antigen bank. Accordingly, our institute has been developing a range of vaccine strains for future antigen bank use. Establishing a domestic antigen bank would enable a rapid response in the event of the introduction of exotic FMDV strains for which vaccination has not yet been implemented.

Unlike SAT 1 and SAT 2, which were historically restricted to Africa but have more recently spread to the Middle East, SAT 3 remains confined to Africa [8]. Nevertheless, the possibility of SAT 3 spreading beyond Africa, including to the Middle East, cannot be excluded considering global livestock trade routes. Therefore, preparation for potential introduction into Korea is warranted. In a previous study, vaccination of pigs with 15 μg/dose of the SAT 3 ZIM-R antigen conferred protective efficacy [9]. However, for commercial application of SAT 3 ZIM-R as an antigen-bank vaccine strain, it is essential to demonstrate high 146S antigen yield, stable scalability, and adequate inactivation kinetics for vaccine manufacturing. The present study was conducted to address these requirements.

In FMD vaccines, the quantity of intact 146S particles rather than pentamers or VP1 is a critical determinant of vaccine quality. Although pentameric antigens and intact virions may induce similar ELISA antibody responses, intact virus particles are known to induce substantially higher neutralizing antibody titers [3,4,5].

Traditionally, FMDV particle content has been measured by sucrose-gradient ultracentrifugation coupled with continuous absorbance monitoring [10,12]. However, such an approach is not practical for studies like ours, which require repeated measurement of multiple samples obtained under different production and inactivation conditions. Therefore, in the present study, FMDV particle content was quantified using an HPLC system equipped with a gel-filtration column, allowing automated and reproducible measurement of virus particle content [11,13,14].

A notable finding of this study was the marked effect of calcium on SAT 3 ZIM-R antigen yield. In the absence of calcium, antigen yields were below 1 μg/mL, whereas supplementation with calcium increased the yield to 7.8 μg/mL in a bioreactor. Although the effect of metal ions on FMDV antigen yield has not been extensively reported, a previous study has suggested that copper ions may have a more favorable effect on FMDV stability than nickel or calcium ions [15], and that calcium supplementation during chromatographic purification can improve virus stability [16]. In hepatitis A virus, calcium treatment has also been reported to enhance receptor binding and increase viral titer [17]. Based on these observations, it is plausible that calcium may enhance SAT 3 ZIM-R binding to the receptor, thereby improving antigen yield. Interestingly, calcium was not required for efficient antigen production of another candidate vaccine strain, SAT 2 ZIM-R [18], indicating that the calcium dependency observed here may be strain-specific and deserves further investigation.

Previous study reported that SAT 3 viruses maintain thermal stability at pH 8.2 or 9.1 [19], whereas another report suggested that SAT 3 exhibits greater thermal stability at pH 8.0 than in other pH ranges [20]. In the present study, SAT 3 ZIM-R showed the highest antigen yields at pH 8.0–8.5, followed by a sharp decrease at pH 9.0. This discrepancy may reflect differences among SAT 3 strains or methodological differences between assays evaluating thermal stability under artificial heating conditions [21] and actual antigen productivity under cell culture conditions.

The antigen yield of 7.8 μg/mL in a bioreactor observed in this study is notably high. Previous studies have generally reported FMDV antigen yields of approximately 1 μg/mL [22,23,24], and in some cases 2–3 μg/mL [25,26,27].

Moreover, earlier reports have suggested that antigen yield is not substantially influenced by production scale, from small bioreactors to ton-scale manufacturing systems [26,28]. Therefore, the recovery of 7.8 μg/mL antigen in the present bioreactor system, which is about three- to eight-fold higher than the more typical 1–3 μg/mL range, highlights its potential economic advantage for large-scale vaccine manufacturing.

BHK-21 cells are widely employed worldwide for FMD vaccine antigen production; however, antigen yield can vary depending on factors such as culture medium composition and additives (e.g., calcium) during virus infection. Furthermore, optimal conditions for antigen production may differ not only between serotypes but also among subtypes within the same serotype, underscoring the importance of strain-specific process optimization. In addition to upstream production variables, antigen stability during downstream processing represents another critical determinant of overall yield. During BEI-mediated inactivation, antigen loss may occur as a result of temperature effects, the chemical impact of BEI, or a combination of both, with the extent of loss varying depending on the specific FMDV strain. The reduction in antigen content appeared to be driven mainly by heat exposure. These findings are in contrast to those reported for serotypes O and A of FMDV, in which heat treatment did not result in a reduction in antigen content [29]. However, it is consistent with previous reports showing that SAT serotypes have lower thermal stability than other FMDV serotypes [30].

A previous study demonstrated that vaccination of pigs with the SAT3 ZIM-R antigen confers protective efficacy against viral challenge [9]. In the present study, despite differences in culture processes between flask- and bioreactor-based production, no significant differences were observed in the immunogenicity of the resulting vaccine antigens in pigs. Following a two-dose immunization regimen, both formulations elicited robust neutralizing antibody responses. According to previous studies [31,32], neutralizing antibody titers in the range of approximately 100–200-fold have been reported to confer up to 95% protection against heterologous viruses. Given that vaccination is typically administered as a two-dose regimen, the observation that neutralizing antibody titers approached 500-fold following the second immunization in the present study strongly suggests the potential for robust protective efficacy. These findings suggest that the mode of antigen production did not adversely affect vaccine efficacy.

## 5. Conclusions

Taken together, this study demonstrated that SAT 3 ZIM-R can be produced efficiently under optimized conditions, particularly in the presence of calcium, while maintaining high antigen yield during scale-up. In addition, effective inactivation was achieved using 2 mM BEI at 26 °C, and the resulting antigen induced robust neutralizing antibody responses in pigs regardless of whether it was produced in flasks or in a bioreactor. These findings indicate that SAT 3 ZIM-R is a promising candidate vaccine strain for the future establishment of a domestic FMD antigen bank in Korea.

## Figures and Tables

**Table 1:** Changes in SAT 3 ZIM-R antigen content after BEI treatment at different temperatures. Residual 146S antigen levels were measured after incubation with the indicated BEI concentrations at 26 °C or 37 °C for the designated times. Values are presented as the mean ± standard deviation.

BEI Concentration	26 °C			37 °C		
	0 h	6 h	24 h	0 h	6 h	24 h
0.0 mM	11.5 ± 0.36	11.2 ± 1.39	10.2 ± 0.36	11.5 ± 0.36	11.2 ± 0.95	8.1 ± 0.51
0.5 mM	11.5 ± 0.36	11.6 ± 0.64	10.8 ± 0.91	11.5 ± 0.36	11.6 ± 0.20	7.9 ± 0.17
1.0 mM	11.5 ± 0.36	11.6 ± 0.21	10.6 ± 0.91	11.5 ± 0.36	11.2 ± 0.44	8.3 ± 0.40
2.0 mM	11.5 ± 0.36	11.5 ± 0.44	9.7 ± 0.85	11.5 ± 0.36	11.1 ± 0.10	8.5 ± 0.29
3.0 mM	11.5 ± 0.36	11.0 ± 0.20	9.1 ± 0.91	11.5 ± 0.36	10.9 ± 0.10	7.2 ± 0.17

## Data Availability

The original contributions presented in this study are included in the article. Further inquiries can be directed to the corresponding author.
